# Histopathology images-based deep learning prediction of prognosis in primary mucinous ovarian carcinoma

**DOI:** 10.3389/fonc.2026.1704217

**Published:** 2026-02-06

**Authors:** Mingyi Zhang, Zhixiang Xia, Ruizhi Liu, Zhaojuan Qin, Hongshuai Li, Jia Xu, Qiongxian Long, Yangmei Shen, Bin Liu, Jiyan Liu

**Affiliations:** 1 Department of Biotherapy, Cancer Center, West China Hospital, Sichuan University, Chengdu, Sichuan, China; 2 Center of Statistical Research, School of Statistics and Data Science, Southwestern University of Finance and Economics, Chengdu, Sichuan, China; 3 School of Medical and Life Sciences, Chengdu University of Traditional Chinese Medicine, Chengdu, Sichuan, China; 4 Department of Obstetrics and Gynecology, West China Second Hospital/West China Women’s and Children’s Hospital, Sichuan University, Chengdu, Sichuan, China; 5 Department of Pathology, Nanchong Central Hospital, the Second Affiliated Hospital of North Sichuan Medical College, Nanchong, Sichuan, China; 6 Department of Pathology, West China Second Hospital/West China Women’s and Children’s Hospital, Sichuan University, Chengdu, Sichuan, China; 7 Key Laboratory of Birth Defects and Related Diseases of Women and Children (Sichuan University), Ministry of Education, Chengdu, Sichuan, China

**Keywords:** deep learning, ovarian cancer, primary mucinous ovarian carcinoma, prognosis, whole-slide images

## Abstract

**Background:**

Accurately predicting the prognosis of primary mucinous ovarian carcinoma (PMOC) remains a significant challenge in gynecologic oncology. This study aimed to develop and validate a deep learning model using histopathological images for precise prognostic prediction and risk stratification in PMOC.

**Methods:**

Histopathological slides of PMOC patients were retrospectively collected and digitized into whole-slide images (WSIs). A graph-based deep learning survival model was established by integrating histological feature extraction, spatial graph construction, and survival prediction through graph neural networks (GNN) combined with Cox proportional hazards modeling. Patients were subsequently stratified into high- and low-risk groups based on model-generated risk scores. The model’s prognostic performance was assessed using Kaplan-Meier analysis and Cox regression. Interpretability was evaluated through GNNExplainer-generated heatmaps.

**Results:**

A total of 80 patients (148 WSIs) were included from three medical centers. The best-performing deep learning model achieved a mean C-index of 0.8254 and stratified patients into high-risk and low-risk groups. Patients in the high-risk group demonstrated significantly shorter overall survival (OS) than those in the low-risk group (log-rank *p* = 7.4 × 10^-8^). Multivariate Cox analysis confirmed AI-based risk stratification as an independent prognostic factor (*p* = 0.000298), exhibiting a higher hazard ratio (HR = 7.974) than both FIGO stage (HR = 5.877) and tumor grade (HR = 4.248). GNNExplainer further visualized key regions associated with the model’s predictions, including infiltrative growth patterns and pronounced nuclear atypia.

**Conclusions:**

This deep learning model offers accurate prognostic predictions from histopathology, presenting a promising tool to improve risk stratification and guide personalized treatment in PMOC.

## 1 Introduction

Primary mucinous ovarian carcinoma (PMOC) is a rare subtype of epithelial ovarian cancer, accounting for approximately 3-5% of cases (1, 2). Although early-stage PMOC generally exhibits a favorable prognosis, advanced-stage disease is often resistant to conventional chemotherapy, resulting in poor clinical outcomes (3). Unlike serous ovarian carcinoma, the most common subtype of ovarian, the optimal treatment strategy for PMOC remains controversial due to its unique biological behavior and the absence of reliable prognostic markers (4–7). This uncertainty in predicting patient outcomes hampers individualized treatment decisions and presents a significant challenge for clinicians.

In 2014, the World Health Organization (WHO) introduced a new histologic classification for PMOC based on tumor growth patterns, dividing it into expansile and infiltrative subtypes (8). According to the ESMO-ESGO guidelines, the invasion pattern informs the decision for adjuvant chemotherapy in stage IA/B PMOC, and a 2022 multicenter study demonstrated that the infiltrative pattern has independent, time-dependent prognostic value, particularly within the first two years post-diagnosis (9, 10). However, conflicting evidence exists; a 2023 study reported that, in multivariate analysis, the infiltrative pattern was not independently associated with poorer progression-free survival (PFS) or overall survival (OS) (11). Moreover, assessing invasion patterns remains inherently subjective, with only moderate interobserver agreement even among expert pathologists (12–14). Although molecular approaches such as immunohistochemistry and transcriptomic profiling have been explored to enhance PMOC prognostic evaluation, these methods generally add limited incremental value and incur additional time and costs in clinical workflows (10, 14).

In contrast, deep learning, a subset of artificial intelligence (AI), has recently shown remarkable potential in pathology image analysis (15). By leveraging advanced neural network architectures such as convolutional neural networks (CNN) and graph neural networks (GNN), deep learning methods can automatically learn and extract complex, subtle patterns from digital histopathology images. These patterns may contain abundant clinically relevant information that has yet to be fully utilized, potentially enabling deep learning models to achieve superior performance in tasks such as cancer detection, subtype classification, and prognosis prediction (16–19). However, existing AI studies in ovarian cancer have predominantly focused on diagnostic classification of serous carcinoma, the most common subtype, often lacking spatial context modeling and offering limited interpretability (20, 21). To date, no studies have applied deep learning techniques that jointly leverage image features and spatial coordinates for prognostic analysis and risk stratification in PMOC, a rare subtype of ovarian carcinoma.

In this study, we developed and validated a deep learning model to predict prognosis in PMOC patients from routine histopathology images. We aimed to provide a rapid, simple, and accurate prognostic tool that meets the unmet need for reliable risk stratification in PMOC, ultimately facilitating personalized treatment decisions and enhancing clinical outcomes.

## 2 Materials and methods

### 2.1 Patient cohort and data collection

This retrospective study was approved by the Ethics Committee of West China Second Hospital/West China Women’s and Children’s Hospital (approval number: 2023125). Patients pathologically diagnosed with PMOC who underwent ovarian resection as initial treatment between 2010 and 2023 were enrolled from three medical centers: West China Second Hospital, Chengdu Shangjin Nanfu Hospital and Nanchong Central Hospital. Patients without available clinical data or hematoxylin and eosin (H&E)-stained tumor slides were excluded. Two experienced gynecological pathologists reviewed and confirmed all cases and excluded potential misdiagnoses, including a comprehensive evaluation of clinicopathological information to rule out metastatic mucinous ovarian carcinoma (MMOC) patients. Finally, one to three representative H&E-stained slides from each patient’s surgical resection specimen were digitized at ×40 magnification using a KF-PRO-400-HI scanner (Ningbo Jiangfeng Bioinformatics Technology Co., Ltd., China) at a resolution of 0.25 μm/pixel, generating whole-slide images (WSIs) in SVS format. OS of patients was defined as the interval from the pathological diagnosis date to death or the last documented follow-up. All patients were followed up for at least two years.

### 2.2 Graph-based deep learning survival analysis

The proposed model integrates graph-based deep learning with survival analysis to predict patient outcomes directly from histopathology images. Each WSI is initially downsampled from ×40 to ×5 magnification and divided into non-overlapping 256 × 256 pixel patches. Background removal was performed on the saturation channel of the HSV image. The saturation map was first smoothed using a median filter (kernel size = 7) to reduce pixel-level noise. OTSU thresholding was then applied to automatically derive an initial binary tissue mask. The mask was refined with a morphological closing operation using a 4×4 square kernel to reconnect fragmented tissue regions that may arise from staining variability. Small isolated components were removed using an area threshold of 100 pixels (scaled to the segmentation level), ensuring that only meaningful tissue regions were retained. For each remaining region, internal holes were examined, and only holes larger than 16 pixels were kept, with at most eight holes preserved to avoid small artifacts being interpreted as tissue cavities. This sequential procedure produced a clean and stable tissue mask suitable for subsequent patch extraction and graph construction. The remaining tissue-containing patches are encoded into 512-dimensional feature vectors using the CONCH model (a vision-language pretrained model specifically optimized for histopathological semantics) (22). These features, along with the spatial coordinates of the corresponding patches, are used to construct a graph structure, where each node represents a patch and edges are defined by k-nearest neighbor (k-NN, k = 8) relationships based on Euclidean distance.

We then selected two widely used GNN architectures, Graph Convolutional Networks (GCN) and Graph Attention Networks (GAT), and combined each with one of four common graph pooling strategies: Kernel Density Estimation (KDE), attention, max, and mean pooling, to perform graph-level feature extraction (23–25). The best-performing combination was selected as the final model backbone. Through multiple rounds of message passing and feature aggregation, the GNN learns a compact 64-dimensional graph-level representation that captures both local morphological details and global tissue architecture. This representation is subsequently passed to a Cox proportional hazards model, where it is linearly combined with learned regression coefficients to generate a patient-specific risk score.

During training, the parameters of both the GNN and the Cox model are jointly optimized by minimizing the negative partial log-likelihood loss derived from observed survival data. Once trained, the model can infer a risk score for new WSIs by extracting the 64-dimensional graph representation and applying the learned Cox regression coefficients.

### 2.3 Risk stratification and survival prediction

To stratify patients into high-risk and low-risk groups, we randomly divided the entire dataset into four non-overlapping subsets of equal size at the patient level, ensuring that WSIs from the same patient were assigned to the same subset. In each round, one subset served as the test set, while the remaining three subsets formed the training set used to build the predictive model.

After training, we first calculated patient-specific risk scores for each patient in the test set. For patients with more than one WSI, the highest risk score was selected as the final patient-specific score. Subsequently, using the risk scores and survival data from the training set, we estimated the baseline survival function according to the Cox proportional hazards framework. With this baseline survival function and each test patient’s risk score, we computed the predicted 1.5-year survival probability for every patient in the test set.

Finally, all patients were classified into high-risk or low-risk groups based on a predetermined survival probability threshold, enabling further analysis of differences in survival outcomes between these groups.

### 2.4 Interpretability of prognostic predictions

To enhance the interpretability of our trained survival prediction model, we utilized GNNExplainer, an interpretability tool specifically designed for graph neural networks (26). For each patient’s WSI, the trained GNN first computed a corresponding risk score. Subsequently, the WSI and trained GNN model were jointly analyzed by GNNExplainer, which identified the contribution of each graph node (patch) toward the computed risk score through iterative analysis of the message passing and aggregation processes. These node-level contributions were then mapped back to their spatial locations on the original WSI, generating a heatmap visualization of the prediction mechanism. The resulting heatmap intuitively highlights tissue regions most critical for prognostication, thereby providing transparent and interpretable insights into the model’s predictions and facilitating clinically informed decision-making.

### 2.5 Statistical analysis

Baseline characteristics between groups were compared using the Student’s t-test or Mann-Whitney U test for continuous variables, and the Pearson chi-square test or Fisher’s exact test for categorical variables. Survival curves were estimated using the Kaplan-Meier method and compared using the log-rank test. Univariate and multivariate Cox proportional hazards models were applied to evaluate the prognostic significance of selected factors. A *p*-value < 0.05 was considered statistically significant. All statistical analyses were performed using Python (SciPy stats module and the lifelines package).

## 3 Results

### 3.1 Total study population and prognostic model

A total of 80 PMOC patients, including 148 WSIs, were collected in this study (**Table 1**). The median age at diagnosis was 40.5 years (range, 13-72), and the BMI was 22.4 kg/m² (range, 15.43-38.99). At diagnosis, most patients (81.25%) were FIGO stage I, while 3.75% and 15.00% were stage II and III, respectively. Regarding tumor differentiation, 75.00% were well-differentiated, 20.00% moderately differentiated, and 5.00% poorly differentiated. Tumor size was ≥10 cm in 70.00% of patients. Preoperative serum tumor marker levels varied widely, with median values of 38.75 IU/mL for CA-125, 34.05 IU/mL for CA-199, and 1.10 IU/mL for CEA.

**Table 1:** Clinicopathologic characteristics of all PMOC patients.

Characteristics	West China Second Hospital	Shangjin Nanfu Hospital and Nanchong Central Hospital	Total
	(n = 63, 78.75%)	(n = 17, 21.25%)	(n=80, 100%)
Age at diagnosis, year			
Median (range)	43.00 (18.00–72.00)	31.00 (13.00–62.00)	40.50 (13.00–72.00)
BMI, kg/m^2^			
Median (range)	22.58 (15.61–38.99)	21.11 (15.43–30.49)	22.40 (15.43–38.99)
FIGO stage			
I	50 (79.37)	15 (88.24)	65 (81.25)
II	1 (1.59)	2 (11.76)	3 (3.75)
III	12 (19.05)	0 (0.00)	12 (15.00)
IV	0 (0.00)	0 (0.00)	0 (0.00)
Grade			
Well-differentiated	47 (74.60)	13 (76.47)	60 (75.00)
Moderately differentiated	12 (19.05)	4 (23.53)	16 (20.00)
Poorly differentiated	4 (6.35)	0 (0.00)	4 (5.00)
Tumor size			
≥ 10 cm	45 (71.43)	11 (64.71)	56 (70.00)
< 10 cm	18 (28.57)	6 (35.29)	24 (30.00)
Tumor markers at diagnosis			
CA-125, IU/mL Median (range)	49.10 (6.50–1483.00)	18.00 (6.00–91.00)	38.75 (6.00–1483.00)
CA-199, IU/mL Median (range)	47.10 (4.60–14829.00)	9.80 (2.20–123.00)	34.05 (2.20–14829.00)
CEA, IU/mL Median (range)	1.10 (0.25–221.00)	1.00 (0.25–3.70)	1.10 (0.25–221.00)
AI-based classification			
Median (range)High-risk patients	12 (19.05)	1 (5.88)	13 (16.25)
Median (range)Low-risk patients	51 (80.95)	16 (94.12)	67 (83.75)

**Figure 1:**
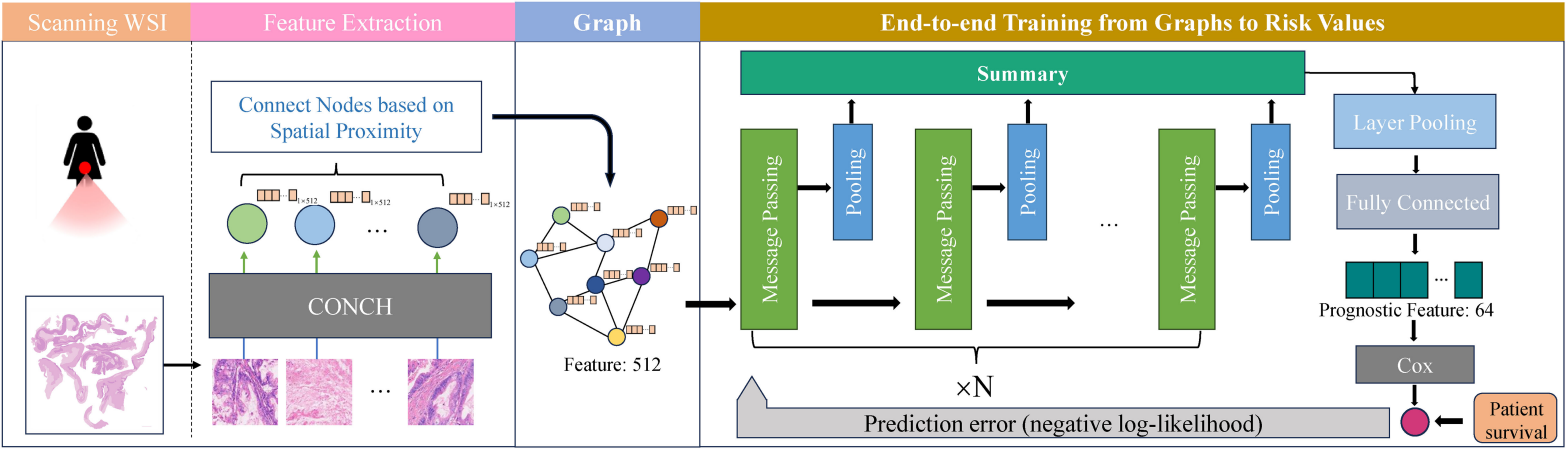
Workflow of the graph-based deep learning model for PMOC prognosis prediction.

As illustrated in **Figure 1**, we constructed a graph-based deep learning model to predict prognosis from WSIs. The pipeline sequentially integrates feature extraction via a pretrained model (CONCH), spatial structure encoding through graph construction and survival risk modeling using a GNN combined with a Cox proportional hazards model. The model generates a patient-specific risk score, allowing for stratification into high- and low-risk groups.

To optimize model performance, we systematically evaluated combinations of two GNN architectures (GCN and GAT) with four pooling strategies (KDE, attention, max, and mean pooling). Among all configurations, the GCN combined with KDE-pooling achieved the highest mean concordance index (C-index) and demonstrated the most consistent performance (**Supplementary Figure 1**). This configuration was therefore selected as the final model backbone, ensuring both accuracy and robustness for clinical prognostic applications.

### 3.2 Comparison between AI-defined risk groups

In our study, the AI model stratified all PMOC patients into two prognostic subgroups: 13 patients (16.25%) in the AI-predicted high-risk group and 67 patients (83.75%) in the low-risk group. Patients classified as high-risk exhibited significantly more advanced FIGO stages at diagnosis compared to the low-risk patients (*p* = 0.020505). Specifically, 46.15% of high-risk patients were diagnosed at FIGO stages II-III, whereas only 13.43% of low-risk patients presented at these advanced stages. Additionally, tumor differentiation grade and tumor size showed statistically significant differences between the two groups (*p* = 0.000014 and *p* = 0.017274, respectively). However, age and preoperative serum tumor markers (CA125, CA199, and CEA) did not differ significantly between the high-risk and low-risk subgroups (**Table 2**).

**Table 2:** Comparison of AI-predicted high-risk and low-risk patients.

Characteristics	High-risk patients	Low-risk patients	*p*-value
	(n = 13, 16.25%)	(n = 67, 83.75%)	
Age at diagnosis, year			0.509933
Median (range)	37.00 (18.00–60.00)	42.00 (13.00–72.00)	
FIGO stage			0.020505
I	7 (53.85)	58 (86.57)	
II	1 (7.69)	2 (2.99)	
III	5 (38.46)	7 (10.45)	
IV	0 (0.00)	0 (0.00)	
Grade			0.000014
Well-differentiated	6 (46.15)	54 (80.60)	
Moderately differentiated	3 (23.08)	13 (19.40)	
Poorly differentiated	4 (30.77)	0 (0.00)	
Tumor size			0.017274
≥ 10 cm	5 (38.46)	51 (76.12)	
< 10 cm	8 (61.54)	16 (23.88)	
Tumor markers at diagnosis			
CA-125, IU/mL Median (range)	49.10 (20.10–221.80)	38.50 (6.00–1483.00)	0.368177
CA-199, IU/mL Median (range)	51.10 (5.40–6993.80)	31.40 (2.20–14829.00)	0.958395
CEA, IU/mL Median (range)	2.00 (0.25–9.10)	1.00 (0.25–221.00)	0.536758

### 3.3 Survival analysis and prognostic validation

**Figure 2:**
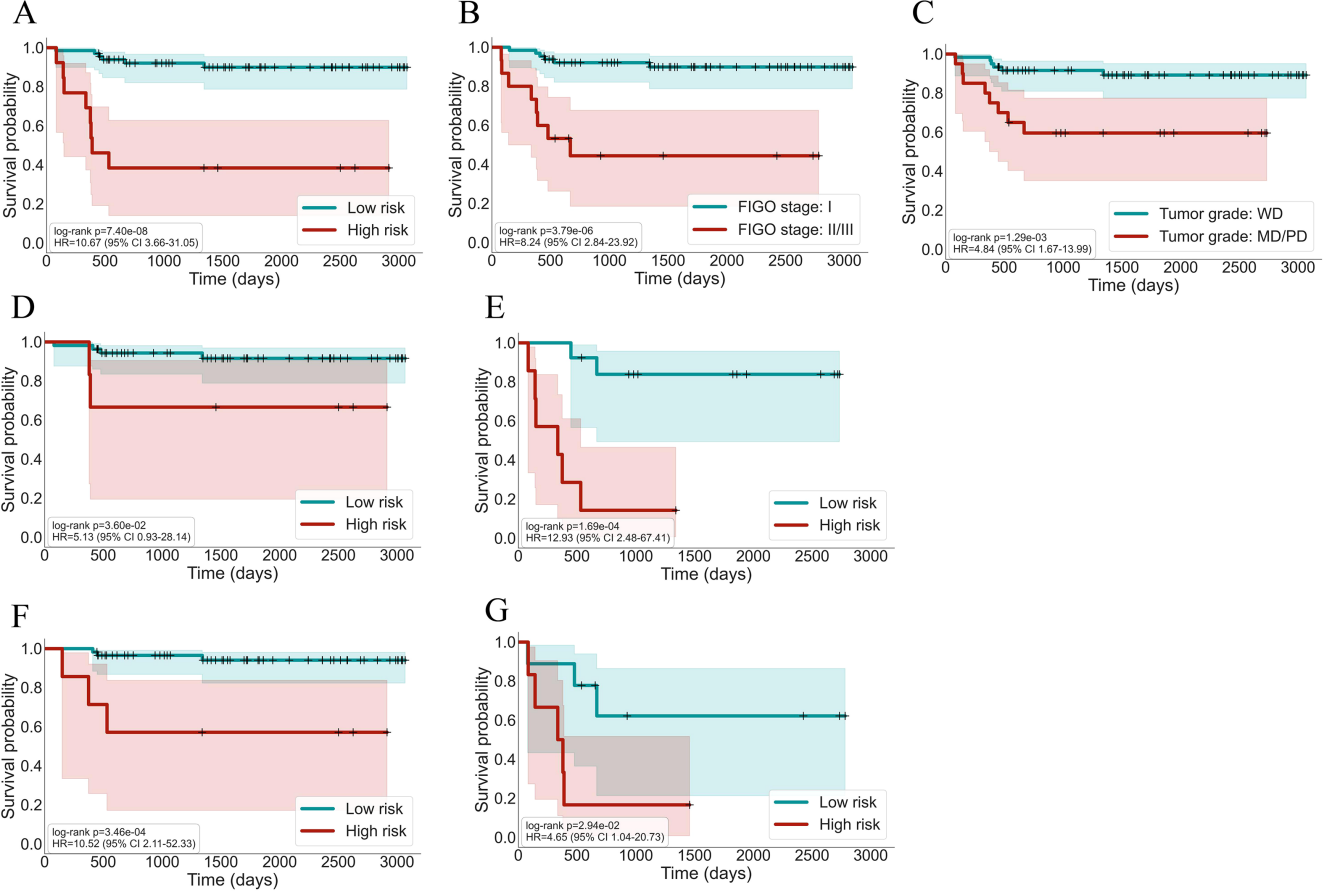
Kaplan-Meier survival curves stratified by different prognostic factors. **(A)** All patients stratified by AI-based risk; **(B)** all patients stratified by FIGO stage; **(C)** all patients stratified by grade; **(D)** FIGO stage I subgroup stratified by AI-based risk; **(E)** FIGO stage II/III subgroup stratified by AI-based risk; **(F)** well-differentiated subgroup stratified by AI-based risk; and **(G)** moderately to poorly differentiated subgroup stratified by AI-based risk.

During a median follow-up of approximately 56 months, a total of 14 death events were observed in the cohort. To evaluate the prognostic value of our AI-based risk stratification model, we performed Kaplan-Meier survival analysis. As shown in **Figures 2A-C**, patients in the AI-defined high-risk subgroup exhibited significantly shorter OS compared to those in the low-risk subgroup (log-rank *p* = 7.4 × 10^-8^). Similarly, patients with advanced FIGO stages (II-III) and those with moderate-to-poor tumor differentiation also demonstrated significantly poorer OS (log-rank *p* = 3.79 × 10^-6^ and 0.00129, respectively).

We further assessed prognostic factors using Cox regression analysis (**Table 3**). Univariate Cox analysis revealed that AI-based risk stratification, FIGO stage, and tumor grade were significant predictors of OS (*p* = 0.000014, 0.000104, and 0.003598, respectively). In contrast, age, invasion patterns and preoperative serum tumor markers were not significantly associated with OS. Subsequent multivariate Cox regression analysis confirmed that AI-based risk stratification remained an independent predictor of OS (*p* = 0.000298). Specifically, patients classified as high-risk by the AI model exhibited a hazard ratio (HR) of 7.974 (95% CI: 2.589–24.567) compared to those in the low-risk group. By comparison, FIGO stage and tumor grade also independently predicted OS, but with relatively lower HRs of 5.877 and 4.248, respectively. Given the limited number of events, we additionally performed L1-penalized (Lasso) Cox regression, which consistently retained the AI-based risk score as a significant predictor across a range of regularization strengths (**Supplementary Figure 2**).

**Table 3:** Univariable and multivariable analyses of OS.

Variables	Univariable analysis		Multivariable analysis	
	HR (95% CI)	*p*-value	HR (95% CI)	*p*-value
Age ≥60y vs. <60y	0.906 (0.203–4.054)	0.897563	–	–
FIGO stage II/III vs. I	8.244 (2.842–23.921)	0.000104	5.877 (1.964–17.581)	0.001536
Invasion patterns (infiltrative vs expansile)	2.849 (0.987–8.224)	0.052864	–	–
Grade (MD/PD vs WD)	4.839 (1.674–13.989)	0.003598	4.248 (1.397–12.914)	0.010791
CA125 (≥50 vs <50 IU/mL)	2.450 (0.820–7.317)	0.108500	–	–
CA199 (≥50 vs <50 IU/mL)	1.430 (0.501–4.079)	0.503447	–	–
CEA (≥1 vs <1 IU/mL)	2.813 (0.784–10.085)	0.112423	–	–
AI-based classification (high vs. low-risk)	10.666 (3.664–31.049)	0.000014	7.974 (2.589–24.567)	0.000298

Moreover, subgroup analyses (**Figures 2D-G**) indicated that even among traditionally favorable patient subgroups, such as those with FIGO stage I or well-differentiated tumors, the AI-defined high-risk patients continued to show significantly shorter OS compared to their low-risk counterparts. Collectively, these findings highlight the model’s robust capability to identify patients with poorer prognosis, even within conventionally low-risk subgroups.

### 3.4 Explainability of prognostic prediction

**Figure 3:**
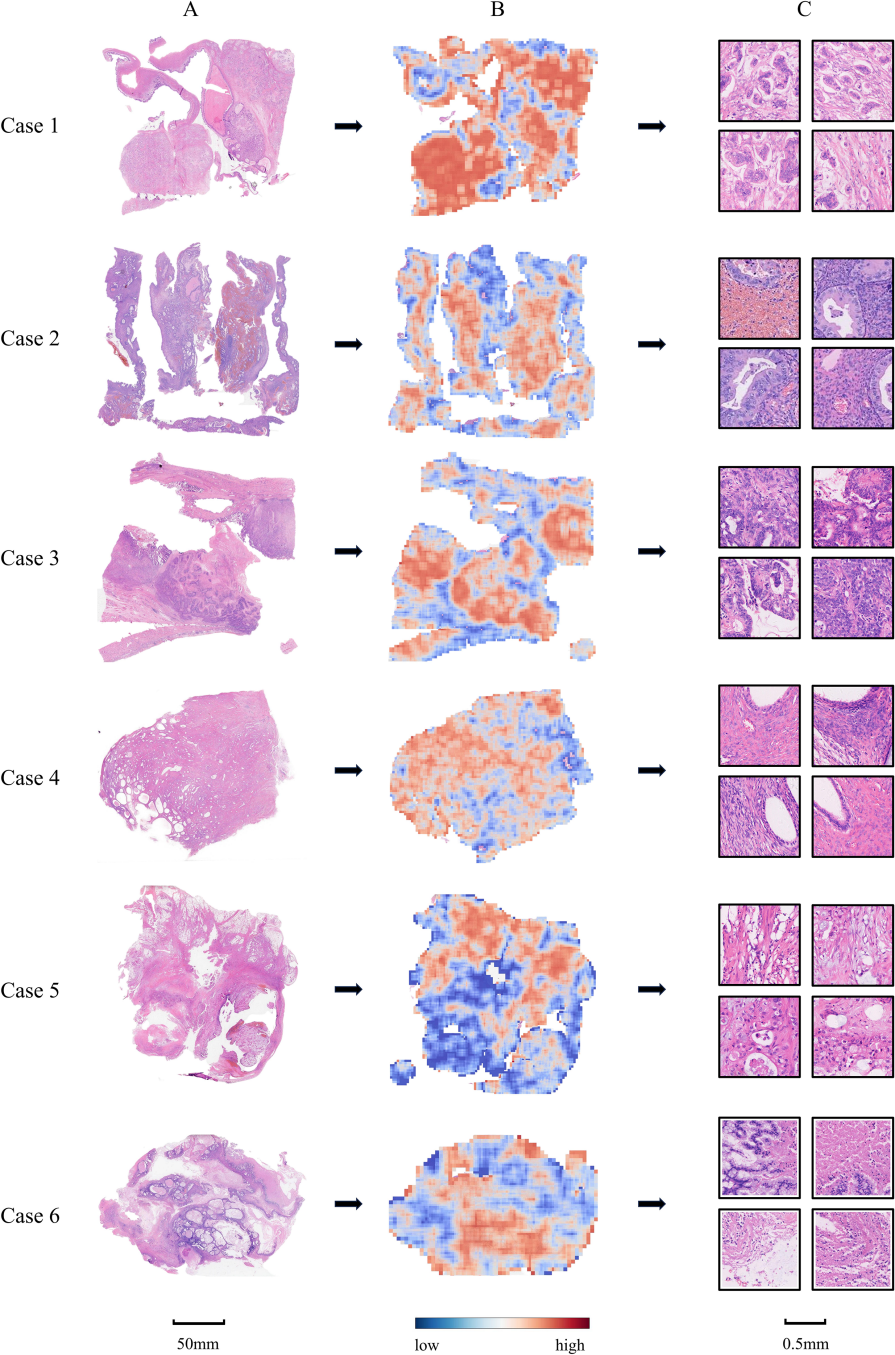
Visual interpretation of model predictions for representative high-risk cases. **(A)** Original H&E-stained slides; **(B)** Corresponding heatmaps generated by GNNExplainer, where red indicates regions with high contribution to risk prediction and blue indicates low contribution; **(C)** Patch-level visualizations of model-attended regions.

To enhance the interpretability of our AI-based prognostic predictions, we employed GNNExplainer to elucidate the model’s decision-making process. Specifically, GNNExplainer identified and visualized the tissue regions within WSIs that had the greatest influence on risk predictions. As illustrated in **Figure 3**, the generated heatmaps highlight the most informative patches, with red areas indicating regions of highest importance. Notably, several high-attention regions overlapped with histological features potentially associated with poor prognosis, such as infiltrative growth patterns and pronounced nuclear atypia.

## 4 Discussion

PMOC is a relatively rare subtype of epithelial ovarian carcinoma with uncertain optimal clinical management. Reliable prognostic biomarkers are urgently needed to facilitate personalized therapeutic decisions. To address this clinical challenge, we developed and validated, for the first time, a deep learning-based prognostic model leveraging routine histopathological images to accurately and objectively stratify PMOC patients into distinct risk groups.

Our model demonstrated promising prognostic performance, clearly distinguishing high-risk from low-risk patients with significantly different OS as confirmed by Kaplan-Meier analysis. Multivariate Cox regression analysis further established AI-based risk stratification as an independent prognostic factor, with the HR of 7.974, exceeding that of conventional indicators such as FIGO stage (HR = 5.877) and tumor differentiation grade (HR = 4.248). Subgroup analyses reinforced the model’s efficacy by identifying patients with poorer prognoses even within traditionally favorable subpopulations, such as those with FIGO stage I disease or well-differentiated tumors. Thus, our model may be a potentially valuable clinical tool, enabling accurate and early identification of high-risk patients who may benefit from enhanced clinical surveillance or treatment intensification.

Previous prognostic studies in PMOC have extensively relied on manually extracted histological features, such as infiltrative growth patterns. Although some studies suggest that infiltrative subtypes independently predict poorer outcomes, conflicting findings highlight the limitations of relying on a single manually extracted morphological feature (11). Additionally, assessment of infiltrative patterns is inherently subjective and exhibits only moderate interobserver consistency, limiting reproducibility and clinical reliability (12–14). By contrast, our graph-based deep learning model comprehensively captures both local morphological features and global structural context from histopathological images, thereby offering an accurate, objective, and reproducible approach to prognostic assessment. Moreover, molecular markers such as fibroblast activation protein (FAP), thrombospondin-2 (THBS2), and transgelin (TAGLN) have been reported to be associated with poor prognosis in patients with PMOC (10, 14). Recent studies have also proposed additional biomarker-based prognostic models in malignancies, further underscoring the rapidly evolving landscape of prognostic research (27–31). However, these markers typically require costly and time-consuming molecular assays and offer limited incremental prognostic value. In contrast, our AI model, which relies solely on routinely available histopathology slides, offers a more practical, efficient, and cost-effective solution suitable for real-world clinical implementation.

Interpretability remains crucial for the clinical acceptance and practical application of AI models. To elucidate the underlying rationale for our model’s predictions, we utilized GNNExplainer, a visualization tool designed for graph-based models. This approach highlighted critical tissue regions that most significantly influenced prognostic outcomes. Notably, several identified regions were found to correspond with potential adverse histological features upon pathological review, such as infiltrative growth patterns and pronounced nuclear atypia. These visual explanations facilitate clinicians’ understanding of AI-derived decisions, enhancing trust and promoting informed clinical judgment.

Nevertheless, several limitations should be acknowledged. First, due to the rarity of PMOC, the sample size was modest, and the current evaluation represents internal rather than external validation. Second, as with other retrospective cohort studies, this work is subject to several methodological threats to validity. These include potential temporal bias arising from changes in diagnostic criteria, treatment strategies, and clinical management over the extended study period; residual confounding due to unmeasured clinical or treatment-related factors that may have influenced survival outcomes; and information bias resulting from variability in tissue processing and staining quality across centers and over time. Third, although our expert pathologists confirmed that some high-attention regions corresponded to adverse histologic features, understanding the specific basis underlying the model’s predictions remains challenging.

Future studies are warranted to extend the present work further. In particular, prospective multi-center validation in larger cohorts will be important to confirm robustness and generalizability. In addition, integrating histopathological features with other available clinical and molecular characteristics or imaging data may further enhance prognostic performance, while continued efforts to clarify the mechanisms underlying model’prediction may improve interpretability and clinical acceptance.

## 5 Conclusions

In summary, we present a novel, interpretable, graph-based deep learning model that enables accurate prognostic prediction and risk stratification in PMOC using standard histopathological images. Our model outperformed traditional prognostic indicators and demonstrated strong potential in identifying high-risk patients who may benefit from intensified monitoring or therapeutic interventions. By eliminating reliance on subjective histological assessments or costly molecular testing, this AI-driven approach may offer a practical and scalable tool for routine clinical use.

## Data Availability

The datasets generated and/or analyzed during the current study are not publicly available due to patient privacy restrictions but are available from the corresponding author on reasonable request. The source code is available at https://github.com/xiazhixiang88/Cancer_SGNN.

## Supplementary material

Supplementary Figure 1 Comparison of C-index distribution of different combinations of GNN architectures and pooling strategies.

Supplementary Figure 2 Effect of regularization strength on Cox proportional hazards model outputs. **(A)** Hazard ratio (HR) of key clinical features (stage, grade, and risk category) across different penalizer values (log scale). The shaded areas represent 95% confidence intervals for HR, and the dashed grey line indicates HR = 1 (no risk association); **(B)** Statistical significance (*p*-value) of each feature across increasing regularization strength (log scale). The dashed red line denotes the conventional significance threshold (*p* = 0.05).

## References

[1] Perren TJ . Mucinous epithelial ovarian carcinoma. Ann Oncol. (2016) 27 Suppl 1:i53–7. doi: 10.1093/annonc/mdw087, PMID: 27141073

[2] Köbel M Kalloger SE Huntsman DG Santos JL Swenerton KD Seidman JD . Differences in tumor type in low-stage versus high-stage ovarian carcinomas. Int J Gynecol Pathol. (2010) 29:203–11. doi: 10.1097/PGP.0b013e3181c042b6, PMID: 20407318

[3] Peres LC Cushing-Haugen KL Köbel M Harris HR Berchuck A Rossing MA . Invasive epithelial ovarian cancer survival by histotype and disease stage. J Natl Cancer Inst. (2019) 111:60–8. doi: 10.1093/jnci/djy071, PMID: 29718305 PMC6335112

[4] Gore M Hackshaw A Brady WE Penson RT Zaino R McCluggage WG . An international, phase III randomized trial in patients with mucinous epithelial ovarian cancer (mEOC/GOG 0241) with long-term follow-up: and experience of conducting a clinical trial in a rare gynecological tumor. Gynecol Oncol. (2019) 153:541–8. doi: 10.1016/j.ygyno.2019.03.256, PMID: 31005287 PMC6559214

[5] Armstrong DK Alvarez RD Bakkum-Gamez JN Barroilhet L Behbakht K Berchuck A . Ovarian cancer, version 2.2020, NCCN clinical practice guidelines in oncology. J Natl Compr Canc Netw. (2021) 19:191–226. doi: 10.6004/jnccn.2021.0007, PMID: 33545690

[6] Kurnit KC Sinno AK Fellman BM Varghese A Stone R Sood AK . Effects of gastrointestinal-type chemotherapy in women with ovarian mucinous carcinoma. Obstet Gynecol. (2019) 134:1253–9. doi: 10.1097/AOG.0000000000003579, PMID: 31764736 PMC7100606

[7] Schlappe BA Zhou QC O’Cearbhaill R Iasonos A Soslow RA Abu-Rustum NR . A descriptive report of outcomes of primary mucinous ovarian cancer patients receiving either an adjuvant gynecologic or gastrointestinal chemotherapy regimen. Int J Gynecol Cancer. (2019) 29:904–9. doi: 10.1136/ijgc-2018-000150, PMID: 31097512 PMC7385730

[8] Kurman R Carcangiu M Herrington C Young R . WHO Classification of Tumours of Female Reproductive Organs. 4th ed. Vol. 6. Lyon: International Agency for Research on Cancer (2014). pp. 9–43.

[9] Colombo N Sessa C du Bois A Ledermann J McCluggage WG McNeish I . ESMO-ESGO consensus conference recommendations on ovarian cancer: pathology and molecular biology, early and advanced stages, borderline tumours and recurrent disease†. Ann Oncol. (2019) 30:672–705. doi: 10.1093/annonc/mdz062, PMID: 31046081

[10] Meagher NS Gorringe KL Wakefield M Bolithon A Pang CNI Chiu DS . Gene-expression profiling of mucinous ovarian tumors and comparison with upper and lower gastrointestinal tumors identifies markers associated with adverse outcomes. Clin Cancer Res. (2022) 28:5383–95. doi: 10.1158/1078-0432.CCR-22-1206, PMID: 36222710 PMC9751776

[11] Lim H Ju Y Kim SI Park JH Kim HS Chung HH . Clinical implications of histologic subtypes on survival outcomes in primary mucinous ovarian carcinoma. Gynecol Oncol. (2023) 177:117–24. doi: 10.1016/j.ygyno.2023.08.013, PMID: 37660413

[12] Genestie C Auguste A Al Battal M Scoazec JY Gouy S Lacroix L . Histological classification of mucinous ovarian tumors: inter-observer reproducibility, clinical relevance, and role of genetic biomarkers. Virchows Arch. (2021) 478:885–91. doi: 10.1007/s00428-020-02939-w, PMID: 33009577

[13] Dundr P Bártů M Bosse T Bui QH Cibula D Drozenová J . Primary mucinous tumors of the ovary: an interobserver reproducibility and detailed molecular study reveals significant overlap between diagnostic categories. Mod Pathol. (2023) 36:100040. doi: 10.1016/j.modpat.2022.100040, PMID: 36788074

[14] Köbel M Kang EY Lee S Terzic T Karnezis AN Ghatage P . Infiltrative pattern of invasion is independently associated with shorter survival and desmoplastic stroma markers FAP and THBS2 in mucinous ovarian carcinoma. Histopathology. (2024) 84:1095–110. doi: 10.1111/his.15128, PMID: 38155475

[15] Echle A Rindtorff NT Brinker TJ Luedde T Pearson AT Kather JN . Deep learning in cancer pathology: a new generation of clinical biomarkers. Br J Cancer. (2021) 124:686–96. doi: 10.1038/s41416-020-01122-x, PMID: 33204028 PMC7884739

[16] Chen PC Gadepalli K MacDonald R Liu Y Kadowaki S Nagpal K . An augmented reality microscope with real-time artificial intelligence integration for cancer diagnosis. Nat Med. (2019) 25:1453–7. doi: 10.1038/s41591-019-0539-7, PMID: 31406351

[17] Korbar B Olofson AM Miraflor AP Nicka CM Suriawinata MA Torresani L . Deep learning for classification of colorectal polyps on whole-slide images. J Pathol Inform. (2017) 8:30. doi: 10.4103/jpi.jpi_34_17, PMID: 28828201 PMC5545773

[18] Skrede OJ De Raedt S Kleppe A Hveem TS Liestøl K Maddison J . Deep learning for prediction of colorectal cancer outcome: a discovery and validation study. Lancet. (2020) 395:350–60. doi: 10.1016/S0140-6736(19)32998-8, PMID: 32007170

[19] Zhang Y Yang Z Chen R Zhu Y Liu L Dong J . Histopathology images-based deep learning prediction of prognosis and therapeutic response in small cell lung cancer. NPJ Digit Med. (2024) 7:15. doi: 10.1038/s41746-024-01003-0, PMID: 38238410 PMC10796367

[20] Farahani H Boschman J Farnell D Darbandsari A Zhang A Ahmadvand P . Deep learning-based histotype diagnosis of ovarian carcinoma whole-slide pathology images. Mod Pathol. (2022) 35:1983–90. doi: 10.1038/s41379-022-01146-z, PMID: 36065012

[21] Bergstrom EN Abbasi A Díaz-Gay M Galland L Ladoire S Lippman SM . Deep learning artificial intelligence predicts homologous recombination deficiency and platinum response from histologic slides. J Clin Oncol. (2024) 42:3550–60. doi: 10.1200/JCO.23.02641, PMID: 39083703 PMC11469627

[22] Lu MY Chen B Williamson DFK Chen RJ Liang I Ding T . A visual-language foundation model for computational pathology. Nat Med. (2024) 30:863–74. doi: 10.1038/s41591-024-02856-4, PMID: 38504017 PMC11384335

[23] Kipf TN Welling M . Semi-supervised classification with graph convolutional networks, in: Proceedings of the International Conference on Learning Representations (ICLR), USA: OpenReview.net. (2017).

[24] Veličković P Cucurull G Casanova A Romero A Liò P Bengio Y . Graph attention networks, in: Proceedings of the International Conference on Learning Representations (ICLR), USA: OpenReview.net. (2018).

[25] Oner MU Lee HK Sung WK . Weakly supervised clustering by exploiting unique class count, in: Proceedings of the International Conference on Learning Representations (ICLR), USA: OpenReview.net. (2019).

[26] Ying R Bourgeois D You J Zitnik M Leskovec J . GNNExplainer: generating explanations for graph neural networks. Adv Neural Inf Process Syst. (2019) 32:9240–51., PMID: 32265580 PMC7138248

[27] Wen D Xiao H Gao Y Zeng H Deng J . N6-methyladenosine-modified SENP1, identified by IGF2BP3, is a novel molecular marker in acute myeloid leukemia and aggravates progression by activating AKT signal via de-SUMOylating HDAC2. Mol Cancer. (2024) 23:116. doi: 10.1186/s12943-024-02013-y, PMID: 38822351 PMC11141000

[28] Wang P Xu S Guo Q Zhao Y . Discovery of PAK2 as a key regulator of cancer stem cell in head and neck squamous cell carcinoma using multi-omic techniques. Stem Cells Int. (2025) 2025:1325262. doi: 10.1155/sci/1325262, PMID: 41311809 PMC12657082

[29] Xu S Chen Z Chen X Chu H Huang X Chen C . Interplay of disulfidptosis and the tumor microenvironment across cancers: implications for prognosis and therapeutic responses. BMC Cancer. (2025) 25:1113. doi: 10.1186/s12885-025-14246-1, PMID: 40597807 PMC12210770

[30] Xu S Liu Y Ma H Fang S Wei S Li X . A novel signature integrated of immunoglobulin, glycosylation and anti-viral genes to predict prognosis for breast cancer. Front Genet. (2022) 13:834731. doi: 10.3389/fgene.2022.834731, PMID: 35432482 PMC9011196

[31] Ke H Li P Li Z Zeng X Zhang C Luo S . Immune profiling of the macroenvironment in colorectal cancer unveils systemic dysfunction and plasticity of immune cells. Clin Transl Med. (2025) 15:e70175. doi: 10.1002/ctm2.70175, PMID: 39934971 PMC11813809

