# Supplementary figures and images for: Histopathology images-based deep learning prediction of prognosis in primary mucinous ovarian carcinoma
Source: Front Oncol. 2026 Feb 6;16:1704217. doi: 10.3389/fonc.2026.1704217 (PMC12921705; doi:10.3389/fonc.2026.1704217)

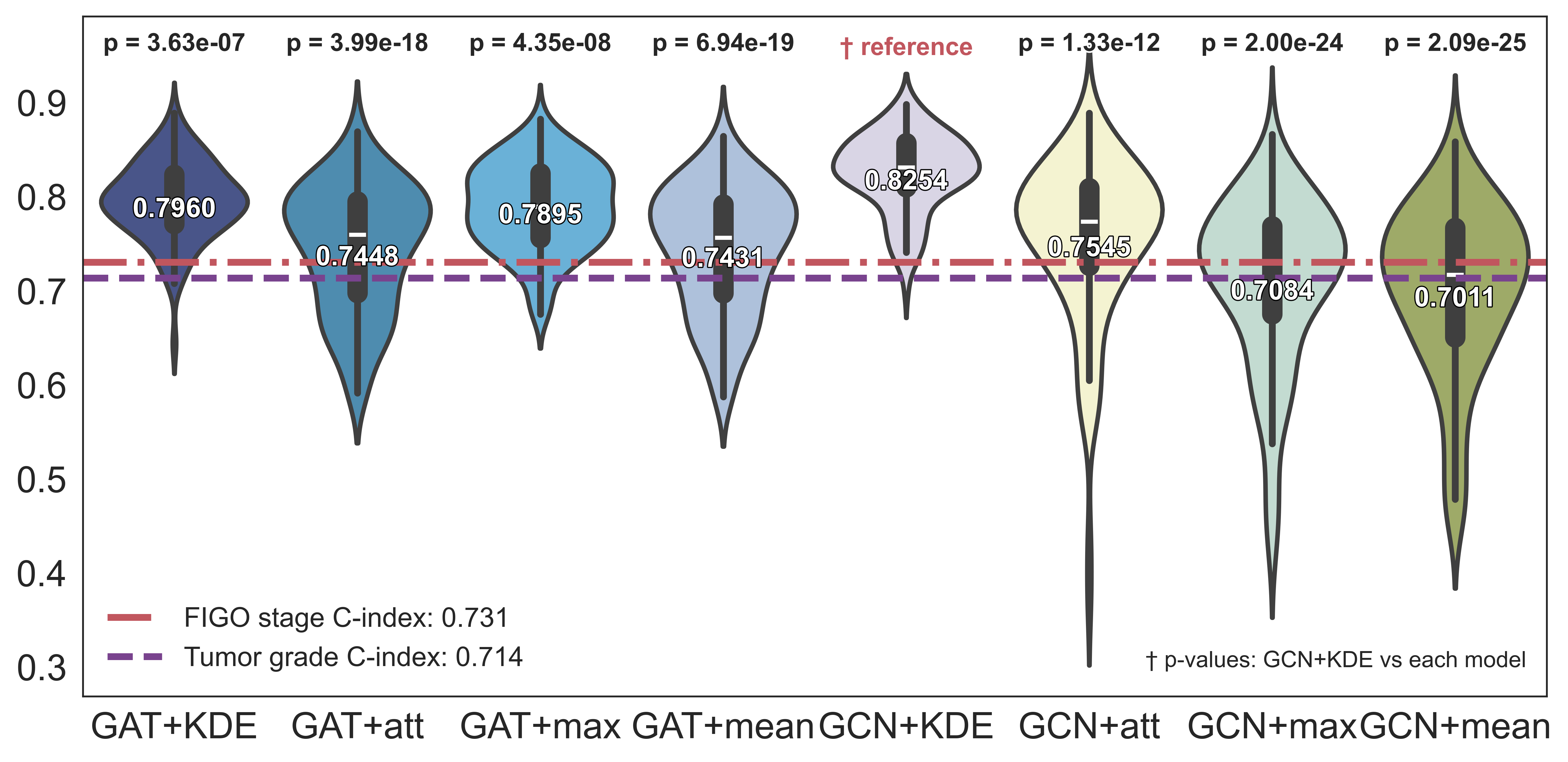

Supplement: Supplementary Figure 1 — Comparison of C-index distribution of different combinations of GNN architectures and pooling strategies. [file Image1.jpeg]

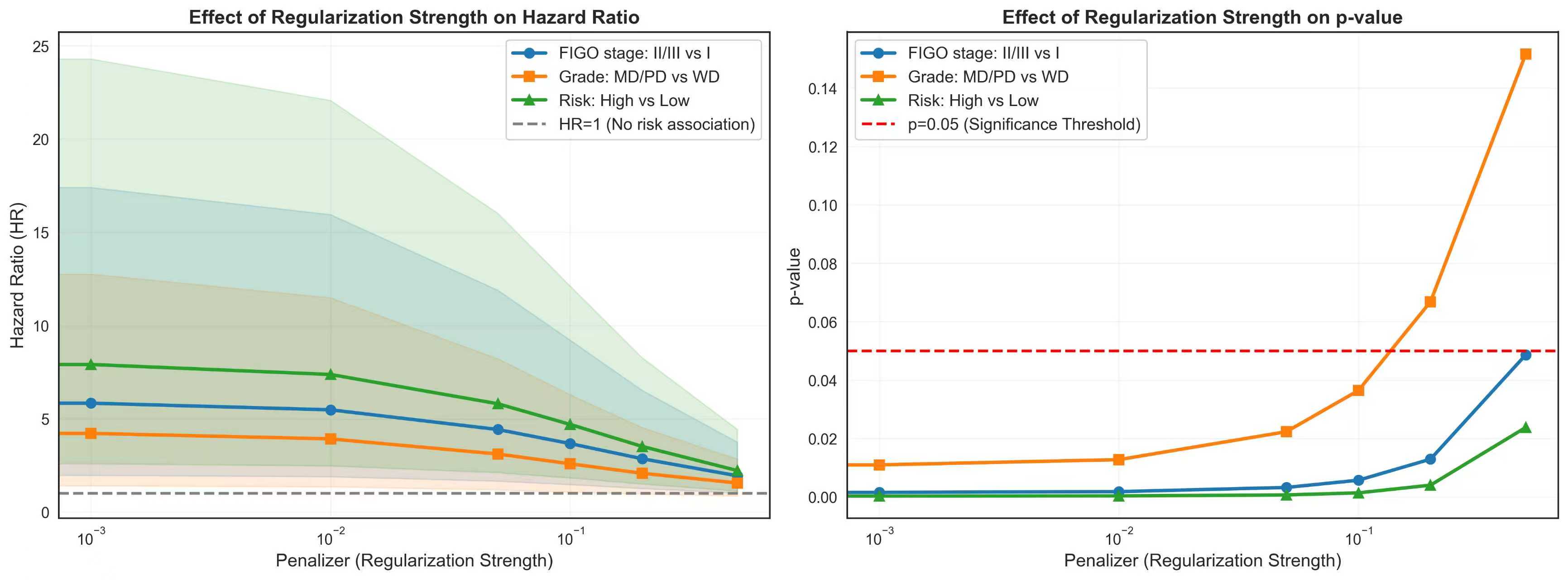

Supplement: Supplementary Figure 2 — Effect of regularization strength on Cox proportional hazards model outputs. (A) Hazard ratio (HR) of key clinical features (stage, grade, and risk category) across different penalizer values (log scale). The shaded areas represent 95% confidence intervals for HR, and the dashed grey line indicates HR = 1 (no risk association); (B) Statistical significance (p-value) of each feature across increasing regularization strength (log scale). The dashed red line denotes the conventional significance threshold (p = 0.05). [file Image2.jpeg]
